# Water-Resistant, Scalable, and Inexpensive Chiral Metal–Organic Framework Featuring Global Negative Electrostatic Potentials for Efficient Acetylene Separation

**DOI:** 10.1021/cbe.3c00093

**Published:** 2024-01-19

**Authors:** Kaiyuan Zhou, Jingjing Zhang, Yuan Geng, Pengfu Gao, Yi Xie, Jinqiao Dong, Yongjia Shang, Yong Cui, Wei Gong

**Affiliations:** † School of Chemistry and Chemical Engineering, Frontiers Science Center for Transformative Molecules and State Key Laboratory of Metal Matrix Composites, Shanghai Jiao Tong University, Shanghai 200240, China; ‡ Key Laboratory of Functional Molecular Solids, Ministry of Education, Anhui Laboratory of Molecule-Based Materials (State Key Laboratory Cultivation Base), College of Chemistry and Materials Science, Anhui Normal University, Wuhu 241002, China; # Department of Chemistry and International Institute for Nanotechnology (IIN), Northwestern University, Evanston, Illinois 60208, United States

**Keywords:** metal−organic framework, acetylene separation, microporous, negative electrostatic potential, chiral

## Abstract

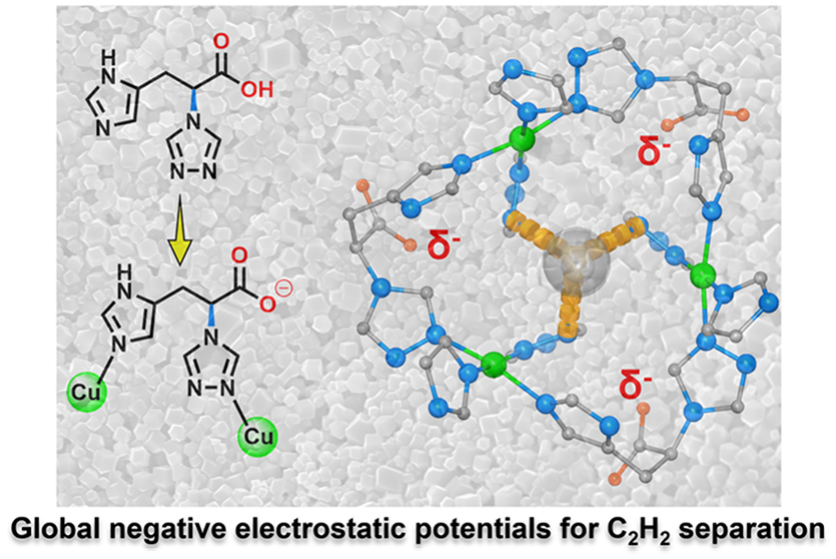

Physical separation of acetylene (C_2_H_2_) from carbon dioxide (CO_2_) or ethylene (C_2_H_4_) on metal–organic frameworks (MOFs) is crucial for achieving high-purity feed gases with minimal energy penalty. However, such processes are exceptionally challenging due to their close physical properties and are also critically restricted by the high cost of large-scale MOF synthesis. Here, we demonstrate the readily scalable synthesis of a highly water-resistant chiral Cu-MOF (**TAMOF-1**) based on an inexpensive proteogenic amino acid derivative bearing rich N/O sites. Notably, the unique coordination in this ultramicroporous MOF has resulted in the generation of rare global negative electrostatic potentials, which greatly facilitate the electrostatic interactions with C_2_H_2_ molecules, thus leading to their efficient separation from C_2_H_2_/CO_2_ and C_2_H_2_/C_2_H_4_ mixtures under ambient conditions. The separation efficiency and mechanism are unequivocally validated by breakthrough experiments and computational simulations. This work not only highlights the pivotal role of creating a negative electro-environment in confined spaces for boosting C_2_H_2_ capture and separation but also opens up new ways of employing cheap amino acid derivatives bearing rich electro-negative N and O sites as organic linkers to constructing high-performing MOF materials for gas separation purposes.

## Introduction

Developing innovative porous solid sorbents for energy-efficient separation of C_2_H_2_ and C_2_H_4_ from C_2_H_2_/C_2_H_4_ and C_2_H_2_/CO_2_ mixtures is crucial for the production of high-purity feed gases and their subsequent polymerization to afford industrially important chemicals.^1−5^ However, due to the very close physical properties of the three gases (Table S1),^6^ it is exceptionally challenging to target solid sorbents that can realize efficient discrimination and separation through pressure swing absorption (PSA) processes. In this context, metal–organic frameworks (MOFs) have been proven to be ideal candidates attributed to their high crystallinity, tunable structures, and customizable pore metrics, which allow us to precisely manipulate and regulate the separation properties at the atomic level.^7−14^

In the past 5–10 years, significant progresses have been made in the field of employing MOFs as sorbents for efficient C_2_H_2_/C_2_H_4_ and C_2_H_2_/CO_2_ separation, resulting in remarkable C_2_H_2_ capture capacity and selectivity by leveraging crystal engineering principles.^15−33^ The dominating principles are the introduction of coordinatively unsaturated metal sites (CUMSs), also known as open metal sites (OMSs), into microporous MOFs for reinforcing framework–C_2_H_2_ interactions through typical side-on coordination between the C≡C bonds and OMSs. For example, Long et al. reported the Fe-MOF-74 featuring high density exposed square pyramidal Fe^2+^ sites capable of binding C_2_H_2_ with strong affinity.^34^ Chen et al. discovered that the increase of OMSs density in MOF-74 can result in the enhanced C_2_H_2_/CO_2_ separation selectivity.^35^ More impressively, Li and Qian et al. developed a novel Hofmann-type MOF bearing sandwich-like binding sites with the two OMSs located exactly opposite to each other, providing optimal pockets for cooperative C_2_H_2_ binding and enabling ultrahigh C_2_H_2_ sorption capacity and C_2_H_2_/CO_2_ and C_2_H_2_/ C_2_H_4_ selectivity.^27^

Recently, the organic functionality engineering has emerged as an intriguing alternative strategy to boost C_2_H_2_/C_2_H_4_ or C_2_H_2_/CO_2_ separation by utilizing the hydrogen acidity difference in C_2_H_2_ (p*K*_a_ = 25) and C_2_H_4_ (p*K*_a_ = 44) and electrostatic potential difference in C_2_H_2_ and CO_2_ (Figure 4a).^15,16,24,25,29,36^ The most representative examples demonstrating the availability of this strategy are those based on MOFs composed of anion pillars, which can create negative pore environments for enhancing electrostatic interactions with the positively-charged H atoms on C_2_H_2_ and impeding the entrance of CO_2_ with negatively-charged ends through electrostatic repulsion.^24^

Our group has a long-standing research interest in constructing chiral MOFs and optimizing the pore microenvironments for enhanced enantioselective discrimination of chiral guests.^9,37−41^ During our exploration on chiral MOF sorbents, we discovered that chiral MOFs constructed from cheap amino acid derived linkers are, in principle, preeminent candidates for realizing efficient C_2_H_2_/C_2_H_4_ and C_2_H_2_/CO_2_ separation because of their microporous nature and rich and readily tunable Lewis-basic N and O moieties, which are expected to be favorable for C_2_H_2_ binding.^42−44^

In this work, we report the transformation of the amino group of l-histidine into a five-membered 1,2,4-triazolyl motif and its mild assembly with Cu^2+^ in water to afford a highly water-resistant ultramicroporous MOF (**TAMOF-1**). Interestingly, the unique metal–ligand coordination has facilitated the complete deprotonation of free carboxylate groups, generating rare global negative potentials capable of attracting C_2_H_2_ molecules and achieving efficient C_2_H_2_/C_2_H_4_ and C_2_H_2_/CO_2_ separation with remarkable gravimetric (88 cm^3^ g^–1^) and volumetric (100.3 cm^3^ cm^–3^) C_2_H_2_ sorption capacity and IAST predicted selectivities at 1 bar (2.1 for equimolar C_2_H_2_/CO_2_ and 4.1 for equimolar C_2_H_2_/C_2_H_4_). Dynamic breakthrough experiments validate its practical C_2_H_2_ separation performances from binary C_2_H_2_/CO_2_ and C_2_H_2_/C_2_H_4_ mixtures. Theoretical calculations reveal that C_2_H_2_ molecules are firmly hydrogen bonded by three N atoms from three 1,2,4-triazolyl motifs related by *C*_3_ symmetry.

## Results and Discussion

### Synthesis and Characterization

**Figure 1:**
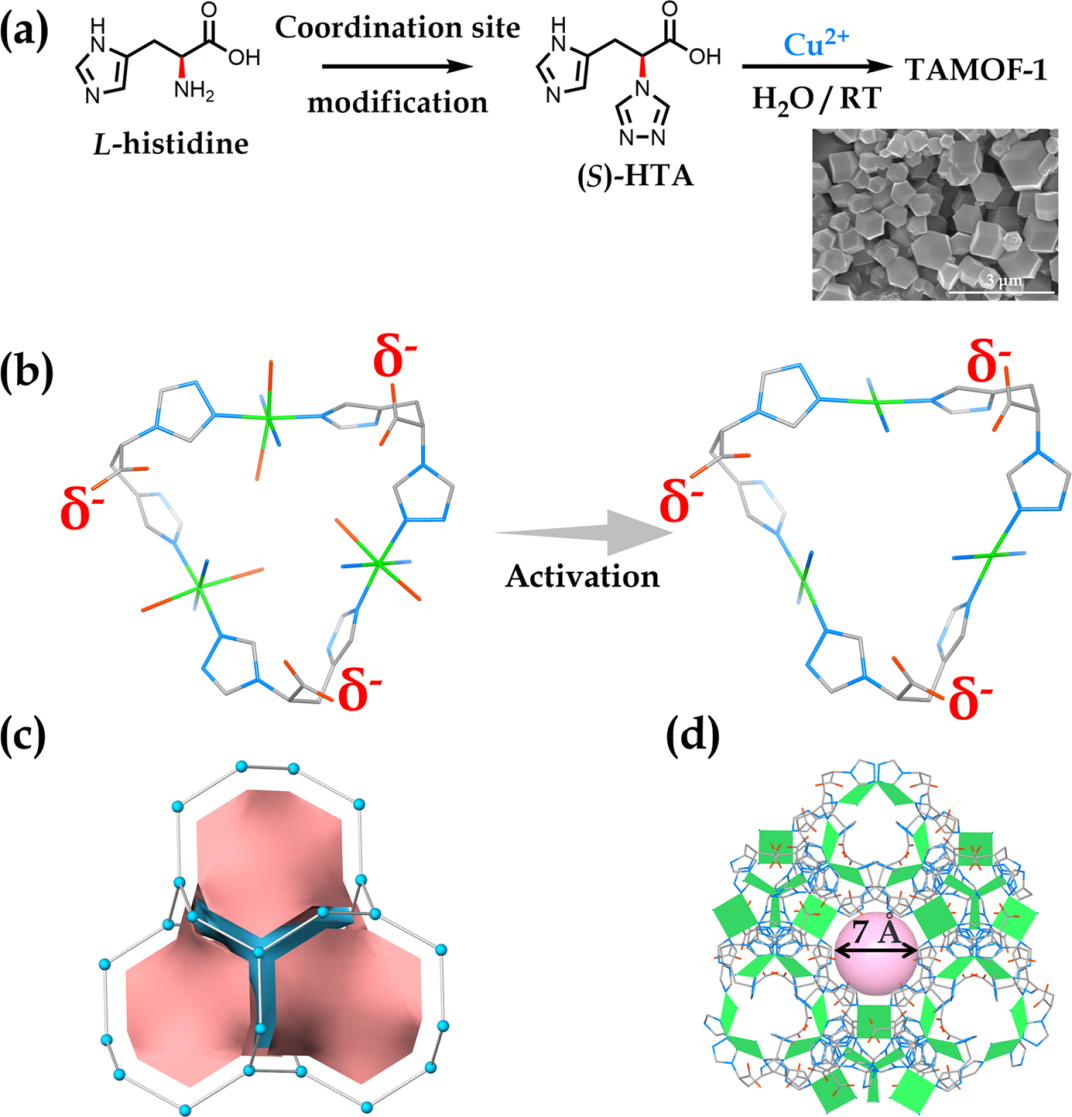
(a) Chemical structure of l-histidine and its further amine modification to afford a triazole group and its assembly with Cu^2+^ in water to form **TAMOF-1** microcrystals with uniform rhombohedral morphology. (b) Triangle pocket in **TAMOF-1** showing the slightly distorted octahedral coordination sphere of Cu^2+^ and its thermal activated structure with terminal water removed. The deprotonated carboxylate groups were indicated by negative potentials, indicating a global negative electro-environment. (c) Simplified intrinsically chiral (10,3)-a **srs** net of **TAMOF-1**, which is shown as the tiling manner. (d) 3D porous structure of **TAMOF-1** viewed along the [1̅11] direction, showing the 1D channels. Color code: green, Cu; blue, N; red, O. Hydrogen atoms have been omitted for clarity.

l-Histidine is a commercially available and inexpensive proteogenic amino acid (∼$60 per kilogram) and is amenable to bind metal ions through rich coordination N/O sites to form microporous MOFs. In order to generate more exposed and accessible Lewis basic sites in the resulted MOFs for enhancing C_2_H_2_ binding affinity, we further transform the labile amino group of l-histidine into a 4*H*-1,2,4-triazole motif through cyclization reaction with *N*,*N*-bis(dimethylaminomethylene)hydrazine (see the Supporting Information for synthetic details) (Figure 1a), with the expectation of further enhancing the structural porosity and robustness. According to the reported procedures by Corella-Ochoa et al.^45^ with slight modifications (see the Supporting Information), we can readily isolate large amount of blue precipitates (**TAMOF-1**) by simply mixing and stirring the derived ligand (HTA) with Cu(CH_3_COO)_2_ in water with high yield (∼75%) (Figure 1a).

**Figure 2:**
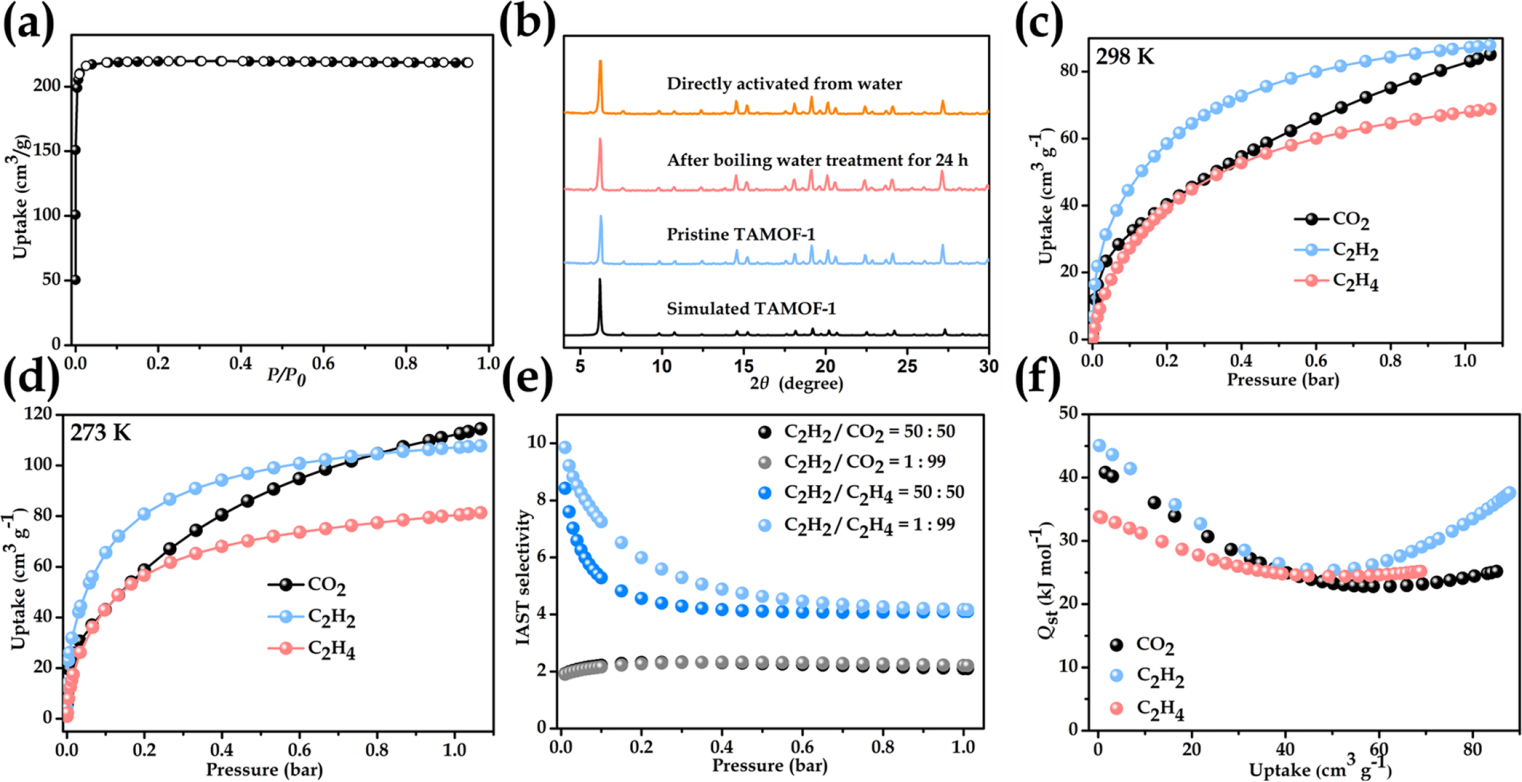
(a) N_2_ adsorption/desorption isotherms of evacuated **TAMOF-1**. (b) PXRD patterns of simulated **TAMOF-1**, freshly prepared **TAMOF-1**, after boiling water treatment for 24 h, and directly thermally activated from water. Experimental single-component sorption isotherms of C_2_H_2_, C_2_H_4_, and CO_2_ at (c) 298 K and (d) 273 K. (e) IAST selectivity curves of **TAMOF-1** for binary C_2_H_2_/C_2_H_4_ and C_2_H_2_/CO_2_ mixtures (v/v = 50/50 and 1/99, respectively). (f) Coverage dependent *Q*_st_ curves of C_2_H_2_, C_2_H_4_, and CO_2_ calculated from adsorption isotherms collected at 273 and 298 K of **TAMOF-1**.

The phase purity of **TAMOF-1** was confirmed by the agreement between the experimental and calculated powder X-ray diffraction (PXRD) patterns (Figure 2b). Scanning electron microscopy (SEM) of freshly prepared **TAMOF-1** microcrystallites indicated a homogeneous rhombohedral morphology with an average size of ∼0.7 μm (Figure 1a). The outstanding water stability of **TAMOF-1** was demonstrated by immersing fresh samples into boiling water for at least 24 h, which showed identical PXRD patterns as the pristine samples (Figure 2b). More impressively, **TAMOF-1** can be directly activated from water at 120 °C without compromising its crystallinity (Figure 2b), which has rarely been observed in MOFs,^46^ especially Cu-based MOFs. Its permanent porosity was evaluated by N_2_ sorption measurements on activated samples at 77 K, giving a typical type-I isotherms and experimental BET area of 730 m^2^ g^–1^ (Figure 2a). Thermogravimetric analysis (TGA) indicates that **TAMOF-1** can be stable until 250 °C (Figure S20).

Careful analysis of the single crystal structure of **TAMOF-1** reveals unusual coordination of Cu ions in which each Cu^2+^ coordinates to two imidazolate (Cu–N instead of Cu-NH), two trizolate, and two water molecules to form a slightly distorted octahedral coordination sphere, leaving the carboxylate groups uncoordinated. Such a coordination environment facilitates the complete deprotonation of carboxylate moieties to conform a neutral framework, thus creating rare global negative electrostatic potentials throughout the framework (Figure 1b). The axially coordinated water molecules are easy to remove under heat because of the Jahn–Teller effect induced elongation of Cu–O bonds, allowing the open Cu sites to be fully accessible (Figure 1b). Topological simplification of **TAMOF-1** gives an intrinsically chiral (10,3)-a **srs** net by considering {Cu(TA)_2_} units as 3-connected nodes (Figure 1c). **TAMOF-1** contains 41% void space and features 3D intersected and helicoidal channels with an apparent pore aperture of ∼0.7 nm, which are accessible to guests (Figure 1d and Figure S2).

### C_2_H_2_, C_2_H_4_, and CO_2_ Sorption and Separation Performance Study

**Figure 3:**
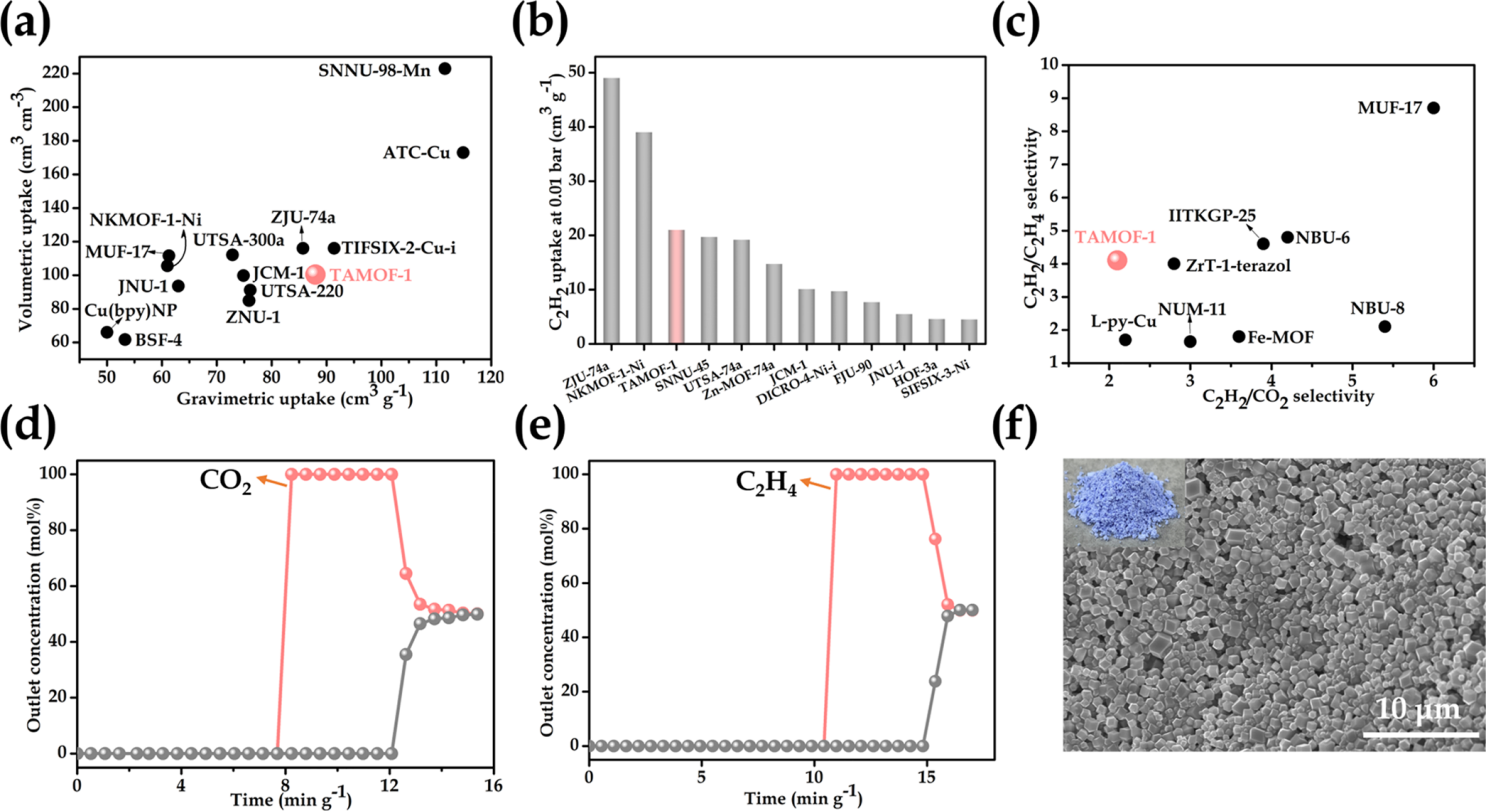
(a) Comparison of gravimetric and volumetric uptake capacities of C_2_H_2_ at 1 bar and 298 K of reported top-performing MOF materials. (b) Comparison of C_2_H_2_ uptake at 0.01 bar and 298 K of **TAMOF-1** with reported top-performing MOF materials. (c) Comparison of C_2_H_2_/C_2_H_4_ and C_2_H_2_/CO_2_ IAST selectivities at 298 K and 1 bar with reported top-performing MOF materials. Experimental column breakthrough curves for (d) an equimolar C_2_H_2_/CO_2_ mixture (298 K, 1 bar) and (e) C_2_H_2_/C_2_H_4_ in an adsorber bed packed with uniform **TAMOF-1** microcrystals. The breakthrough experiments were performed at a flow rate of 2 mL min^–1^, the points are experimental data, and the lines are drawn to guide the eye. (f) SEM image of gram scale synthesized **TAMOF-1** microcrystals. The inset is the picture of **TAMOF-1** powders.

The permanent porosity and framework robustness, uniform distribution of polar groups (free carboxylic acid, triazole, and imidazole), and most importantly, the global negative electrostatic potentials in **TAMOF-1**, have inspired us to investigate its ability to discriminate C_2_H_2_, C_2_H_4_, and CO_2_ and target efficient separation. We first measured the single-component equilibrium isotherms of C_2_H_2_, C_2_H_4_, and CO_2_ at 298 K. As shown in Figure 2c, the C_2_H_2_, C_2_H_4_, and CO_2_ uptakes at 1 bar are comparable, giving gravimetric capacities of 88 (volumetric capacity: 100.3 cm^3^ cm^–3^), 69, and 85 cm^3^ g^–1^, respectively. Notably, the gravimetric and volumetric uptake capacities of C_2_H_2_ at 1 bar and 298 K are among the best-performing MOF sorbents (Figure 3a). When we look at the isotherms at the low-pressure region (0–0.01 bar) (Figure S5), the adsorption capacity of C_2_H_2_ (22 cm^3^ g^–1^) is significantly higher than C_2_H_4_ (6.5 cm^3^ g^–1^) and CO_2_ (14 cm^3^ g^–1^) and is also superior than most benchmark MOF sorbents (Figure 3b), suggesting stronger interactions of C_2_H_2_ molecules with the inner pore surface of **TAMOF-1**. We performed a second cycle of sorption isotherms for C_2_H_2_, C_2_H_4_, and CO_2_ (Figure S15), which demonstrate the great reproductivity of **TAMOF-1** in gas adsorption. To evaluate the binding energies between the framework with the three gases at low coverage, we further collected sorption data at 273 K (Figure 2d) and calculated the coverage-dependent isosteric heats of adsorption (*Q*_st_) based on the Virial equation (Supporting Information). As shown in Figure 2f, the experimental *Q*_st_ values for C_2_H_2_, C_2_H_4_, and CO_2_ at nearly zero coverage are estimated to be 45, 34, and 41 kJ/mol, respectively, indicative of stronger binding affinity of C_2_H_2_ than C_2_H_4_ and CO_2_, which is consistent with the single-component gas sorption isotherms.

To predict the potential of **TAMOF-1** for practical C_2_H_2_/C_2_H_4_ and C_2_H_2_/CO_2_ separation, we calculated the ideal adsorbed solution theory (IAST) selectivity for both 50:50 and 1:99 binary gas mixtures. As shown in Figure 2e, the IAST selectivities for equimolar C_2_H_2_/C_2_H_4_ and C_2_H_2_/CO_2_ mixtures are 4.1 and 2.1, respectively, at 298 K and 100 kPa, which are comparable to the reported C_2_H_2_-selective MOF materials (Figure 3c). For 1:99 binary gas mixtures, the IAST selectivities at 298 K and 100 kPa are almost unchanged.

**Figure 4:**
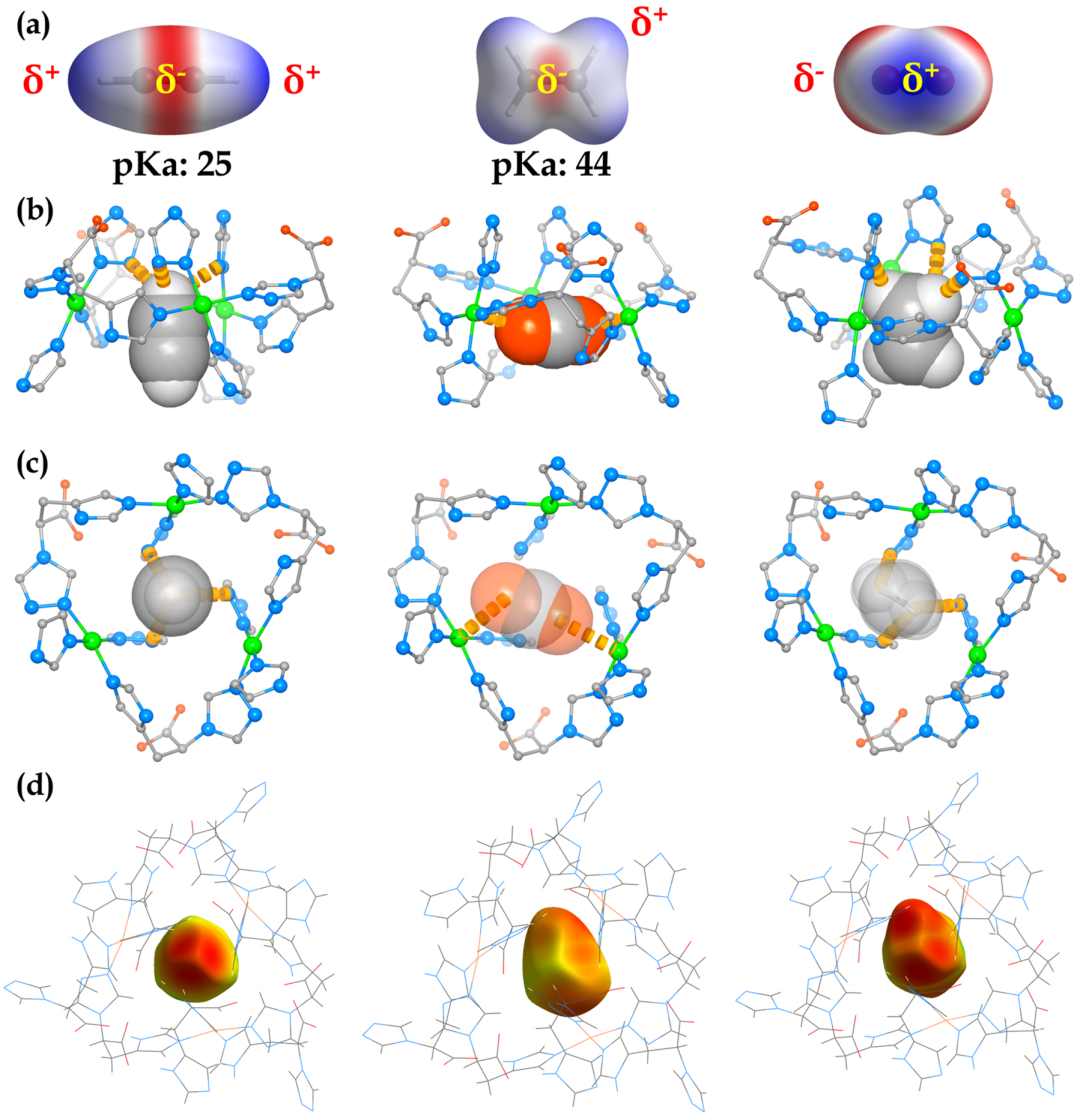
(a) Molecular electrostatic potential (MESP) analysis of C_2_H_2_, C_2_H_4_, and CO_2_. Blue and red colors represent the positive and negative part of MESP, respectively. (b) and (c) DFT-optimized host–guest structures showing the preferential adsorption sites and the interactions of C_2_H_2_, C_2_H_4_, and CO_2_ within the narrow pockets of **TAMOF-1** viewed from the *a* and *c* axes, respectively. The C–H···N and O···Cu interactions are highlighted in yellow dashed lines. (d) Hirshfeld surface analyses of C_2_H_2_, C_2_H_4_, and CO_2_ mapped with *d*_i_, highlighting their contacts with the pore surface (red colored regions indicate contact distances shorter than the sum of vdW radii).

In light of the structural rigidity of **TAMOF-1**, we modeled the preferential locations of C_2_H_2_, C_2_H_4_, and CO_2_ in the porous framework using grand canonical Monte Carlo (GCMC) simulations (see the Supporting Information for simulation details) with the aim of microscopically understanding the adsorption mechanism. The simulated adsorption isotherms for C_2_H_2_, C_2_H_4_, and CO_2_ were in good agreement with the experimental ones (Figure S6). The simulated gas loaded structures at different pressures evidently showed the preferential binding sites of respective gas molecules inside the channels (Figures S7–S12). Moreover, we observed distinct C_2_H_2_–C_2_H_2_ intermolecular interactions (C_2_H_2_^δ+^···^δ−^C_2_H_2_) in the C_2_H_2_-loaded structure, which should have contributed to the dense packing of C_2_H_2_ molecules in the channel (Figure S8). To further understand and visualize the precise molecular interactions of C_2_H_2_, C_2_H_4_, and CO_2_ in **TAMOF-1**, we performed first-principles density functional theory (DFT) calculations to optimize the host–guest structures. As shown in Figure 4b,c, the primary binding sites of C_2_H_2_, C_2_H_4_ are both located at the triangle pockets. Specifically, the C_2_H_2_ molecule was firmly trapped and fixed by three-fold *C*_3_ symmetric C_2_H_2_^δ+^···^δ−^N (triazole) and ^δ−^C_2_H_2_···^δ+^Cu interactions with a H–N distance of 2.543 Å and a C–Cu distance in the range 3.840–4.001 Å. In contrast, the C_2_H_4_ molecule formed similar C_2_H_4_^δ+^···^δ−^N (triazole) interactions but with apparently lower symmetry and weaker binding affinity, as reflected by the calculated binding energy (36.4 kJ/mol) compared with C_2_H_2_ (42.7 kJ/mol). The observed stronger binding energy of C_2_H_2_ than C_2_H_4_ can be ascribed to the higher hydrogen acidity of C_2_H_2_ (Figure 4a). In sharp contrast, due to the electrostatically negative nature, CO_2_ molecules were found to be located near one side of the triangle pocket, forming relatively strong CO_2_^δ−^···^δ+^Cu interactions with Cu–O distances of 2.854 and 2.995 Å, respectively. The derived static binding energy of CO_2_ was calculated to be 37.2 kJ/mol, and the binding energy trend was in agreement with the experimental *Q*_st_ values.

Hirshfeld surface analysis was further performed to more clearly visualize the host–guest interactions by mapping *d*_i_ (distance from a point on the Hirschfeld surface to the nearest nuclei inside the surface) onto the Hirshfeld surface of the confined C_2_H_2_, C_2_H_4_, and CO_2_ using Crystal Explorer.^47^ As shown in Figure 4d, the intense red regions showed as distinct *C*_3_ symmetry indicated strong contacts of C_2_H_2_^δ+^···^δ−^N (triazole) while C_2_H_4_ and CO_2_ showed unsymmetric contacts and moderate interactions. The host–guest interaction components in gas-loaded structures were then analyzed by the derived 2D fingerprint plots (Figure S13). The primary contacts (bright blue spike) were defined to be C–H···N in both C_2_H_2_ and C_2_H_4_, which are mainly attributed to the contributions from C_2_H_2_^δ+^···^δ−^N interactions (14% and 15.2% for C_2_H_2_ and C_2_H_4_, respectively). For CO_2_, the two bright blue spikes were assigned to the CO_2_^δ−^···^δ+^Cu and CO_2_^δ−^···^δ+^H (C–H) contacts account for 8.3% and 53.3% of the overall interactions, which are obviously different with that observed in C_2_H_2_ and C_2_H_4_. These molecular observations thus validate our hypothesis that the rich Lewis basic N sites and global negative potentials in **TAMOF-1** have dictated preferential C_2_H_2_ sorption.

Transient breakthrough experiments of equimolar C_2_H_2_/C_2_H_4_ and C_2_H_2_/CO_2_ binary mixtures for **TAMOF-1** were also carried out to further corroborate their practical separation performances under ambient conditions. As shown in Figure 3d,e, clean and sharp separations were observed. Weakly bonded C_2_H_4_ and CO_2_ were shown to firstly elute through the bed and then C_2_H_2_ started to breakthrough when the adsorbents got saturated, and the outlet gas stream quickly reached equimolar concentrations. These results distinctly demonstrated the efficiency of **TAMOF-1** as a potential C_2_H_2_ selective separation material with minimal energy penalty. We also performed a second cycle of breakthrough experiments (Figures S18 and S19), which demonstrate the good recyclability and durability of **TAMOF-1**. Moreover, we demonstrated that **TAMOF-1** can be easily synthesized at the gram scale by straightforward mixing and stirring the precursors in water, which is highly energy-efficient and environmentally friendly, highlighting the huge potential of **TAMOF-1** for industrial separation technologies. The phase purity, crystallinity, and uniform morphology of the samples are confirmed by PXRD experiments (Figure S14) and SEM image (Figure 3f).

## Conclusions

In summary, we reported the exploration of an amino acid derived ligand for the construction of highly water-resistant MOF material (**TAMOF-1**) and demonstrated its efficient separation toward C_2_H_2_/C_2_H_4_ and C_2_H_2_/CO_2_ mixtures. In contrast to traditional C_2_H_2_ selective materials that typically featured rich OMSs, the unusual coordination in **TAMOF-1** has resulted in the generation of global negative potentials in the 3D intersected channels for preferential discrimination of C_2_H_2_, leading to high gravimetric and volumetric C_2_H_2_ uptake and remarkable separation selectivities. Extensive modeling studies revealed that the introduction of the 1,2,4-triazolyl motif has contributed significantly to the optimal binding of C_2_H_2_. Moreover, we also demonstrated that **TAMOF-1** can be directly activated from water by heat treatment without compromising the crystallinity, indicative of its outstanding water stability. Besides, **TAMOF-1** can be readily synthesized on the gram scale in water and at room temperature with very low cost, suggesting its potential in practical applications. Our work not only highlights the great potential of employing cheap amino acid derivatives bearing rich electronegative N/O sites to assemble ultramicroporous MOF materials for efficient C_2_H_2_ separation but also provides a new perspective to design and modify organic ligands with the aim of fine-tuning MOF topologies and microenvironments for versatile applications.

## Supporting Information Available

Calculations and computational details, general procedures, synthetic details, table of physical properties, and figures of structures and surfaces, adsorption and desorption isotherms and gas sorption isotherms, GCMC-simulated and experimental isotherms, GCMC simulated adsorption structures, 2D fingerprint plot, PXRD patterns, single-component isotherms, second cycle breakthrough curves, TGA, and fitting results of *Q*_st_ and isotherms (PDF)
