# Supplementary material for: Water-Resistant, Scalable, and Inexpensive Chiral Metal–Organic Framework Featuring Global Negative Electrostatic Potentials for Efficient Acetylene Separation
Source: Chem Bio Eng. 2024 Jan 19;1(4):349–56. doi: 10.1021/cbe.3c00093 (PMC11835167; doi:10.1021/cbe.3c00093)
Supplement: Supplementary file 1 — be3c00093_si_001.pdf [file be3c00093_si_001.pdf]

Supporting Information

Water-Resistant, Scalable, and Inexpensive Chiral Metal-Organic Framework Featuring Global Negative Electrostatic Potentials for Efficient Acetylene Separation

Kaiyuan Zhou,[†,‡,§] Jingjing Zhang,[†,§] Yuan Geng,[†] Pengfu Gao,[†] Yi Xie,[#] Jinqiao Dong,[†] Yongjia Shang,[‡] Yong Cui[†], and Wei Gong*,[†]

[†]School of Chemistry and Chemical Engineering, Frontiers Science Center for Transformative Molecules and State Key Laboratory of Metal Matrix Composites, Shanghai Jiao Tong University, Shanghai 200240, China

[‡]Key Laboratory of Functional Molecular Solids, Ministry of Education, Anhui Laboratory of Molecule-Based Materials (State Key Laboratory Cultivation Base), College of Chemistry and Materials Science, Anhui Normal University, Wuhu 241002, China

[#]Department of Chemistry and International Institute for Nanotechnology (IIN), Northwestern University, Evanston, Illinois 60208, United States

Corresponding author E-mail: gongwei_2014jd@sjtu.edu.cn

Fitting of pure component isotherms

The experimentally collected isotherms for C_2H_2, C_2H_4, and CO_2 at 273 K, and 298 K in **TAMOF-1** were fitted with the dual-site Langmuir-Freundlich equation.

$$q = q_{A,sat}\frac{b_A p}{1 + b_A p} + q_{B,sat}\frac{b_B p}{1 + b_B p} \quad (1)$$

The Langmuir parameters for each site is temperature-dependent,

$$b_A = b_{A0} exp\left(\frac{E_A}{RT}\right); b_B = b_{B0} exp\left(\frac{E_B}{RT}\right) \quad (2)$$

Calculation of isosteric heat of adsorption (Q_{st})

The Clausius-Clapeyron equation was employed to calculate the heat of adsorption:

$$Q_{st} = RT^2\left(\frac{\partial ln\, p}{\partial T}\right)_q \quad (3)$$

was determined using the pure component isotherm fits using the Virial equation.

IAST calculations of adsorption selectivity

For the separation of a binary mixture of components C_2H_2/C_2H_4 and C_2H_2/CO_2, the adsorption selectivity is defined by

$$S_{ads} = \frac{q_1/q_2}{y_1/y_2} \quad (4)$$

In equation (4), the q_1, and q_2 represent the molar loadings of C_2H_2/C_2H_4 and C_2H_2/CO_2, expressed in mol kg^{-1}, within the MOF that is in equilibrium with a bulk fluid mixture with mole fractions y_1, and $y_2 = 1\text{-}y_1$. The molar loadings, also called gravimetric uptake capacities, are usually expressed with the units mol kg^{-1}. The IAST calculations of 50/50 mixture adsorption taking the mole fractions $y_1 = 0.5$ and $y_2 = 1\text{-}y_1 = 0.5$ for a range of pressures up to 101 kPa and 298 K were performed.

Computational details

All the calculations were performed in the Material Studio 2019 package (BIOVIA, Dassault Systèmes, Materials Studio 2019, Dassault Systems, San Diego, 2018.).

GCMC simulation:

The crystal structure of **TAMOF-1** was taken as initial geometry for further computational calculations. The partial charge of frameworks was taken from Mulliken charge calculated from DFT and considered as rigid in the simulations. The charges of gas molecules were derived from ESP charge calculated in $Dmol^3$ module using B3LYP functional. The simulation boxes consisted of 1 × 2 × 2 = 4 unit cells and universal forcefield (UFF) was used, in which the interaction energy between gas molecules and frameworks were computed through the Coulomb and Lennard-Jones 6-12 (LJ) potentials. The cut-off radius was chosen as 15.5 Å for the LJ potential and the long-range electrostatic interactions were handled by the Ewald summation method, with a Buffer width of 0.5 Å and accuracy of 1×10^{-5} kcal mol^{-1}.The equilibration steps and the production steps were set as 1×10^6 and 1×10^7, respectively. The saturation uptake of C_2H_2 was calculated at 195 K and 1 bar using universal forcefield. The equilibration steps and the production steps were set as 1×10^6 and 5×10^6, respectively, to ensure the equilibration. The result indicated that 4 C_2H_2 molecules were adsorbed per unit cell. To investigate the host-guest configuration, simulated annealing calculations were performed for 4 C_2H_2 molecules within a unit cell, which started from an initial temperature of 1×10^5 K, followed by 1.0×10^6 Monte Carlo steps. The final temperature of the system was 100 K.

DFT calculation:

The static binding energy was calculated using first-principle density funtional theory (DFT) in the CASTEP code.[1] The generalized gradient approximation (GGA) with the Perdew-Burke-Ernzerhof (PBE) functional[2] and on-the-fly generated ultrasoft pseudopotentials[3] were used. Grimme (G06) semiempirical methods to describe the long-range van der Waals interactions. A cutoff energy of 450 eV and a 2 × 2 × 1 *k*-point mesh was found to be enough for the total energy to converge within 1×10^{-5} eV atom $^{-1}$. Full geometry optimizations were performed on the structures loaded with one C_2H_2, C_2H_4, or CO_2 molecules. The static binding energy was calculated: $\Delta E = E(\mathrm{MOF}) + E(\mathrm{gas}) - E(\mathrm{MOF} + \mathrm{gas})$.

Synthesis of TAMOF-1

Ligand synthesis: *N*, *N*-bis(dimethylaminomethylene)hydrazine (5 g) and *L*-histidine (2.46 g, 0.02 mol) were mixed in 120 mL of EtOH and refluxed with stirring for 48 h. The resulted solution was filtered off and the solvent was removed under reduced pressure to afford an orange gel that was carefully washed with EtOH to yield white precipitates, that was collected by filtration, washed with minimal EtOH, and dried in air.

Large single crystals: $Cu(CH_3COO)_2 \cdot H_2O$ (0.024 g) was dissolved in 1 mL of H_2O, and the resulting solution was slowly added to a 1 mL aqueous solution of HTA (0.05 g). The solution was stirred for 1 min, allowing for the formation of homogeneous clear solution. The solution was left undisturbed for 24 h and large polyhedral crystals were formed and collected by filtration and washed with H_2O.

Gram scale synthesis of microcrystals: $Cu(CH_3COO)_2 \cdot H_2O$ (0.96 g) was dissolved in 100 mL of H_2O, and the resulting solution was slowly added to a 100 mL aqueous solution of HTA (2 g). The solution was stirred for about 1 hour, allowing for the complete formation of blue precipitates, which were identified as pure polycrystalline powder of TAMOF-1. Yield: 75%.

Table S1. Physical properties of C_2H_2, C_2H_4, and CO_2.[4]

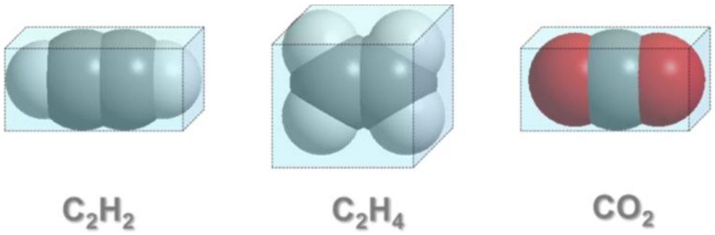

Compound	Carbon dioxide (CO_2)	Acetylene (C_2H_2)	Ethylene (C_2H_4)
Dimensions (Å^3)	3.18×3.33×5.36	3.32×3.34×5.70	3.28×4.18×4.84
Polarizability (Å^3)	2.91	3.33-3.93	4.25
Kinetic Diameter (Å)	3.3	3.3	3.9
Boiling point (K)	194.7	188.4	169.4

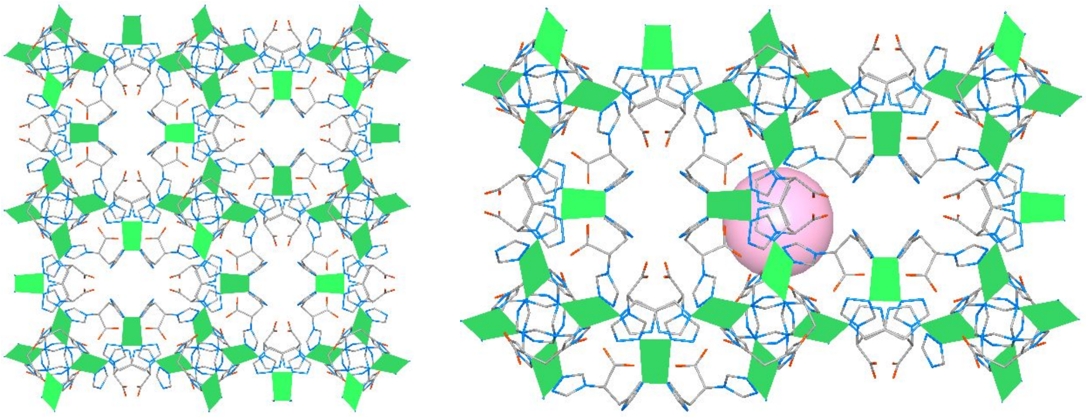

Figure S1. 3D framework structure of **TAMOF-1** viewed along the *c* axis.

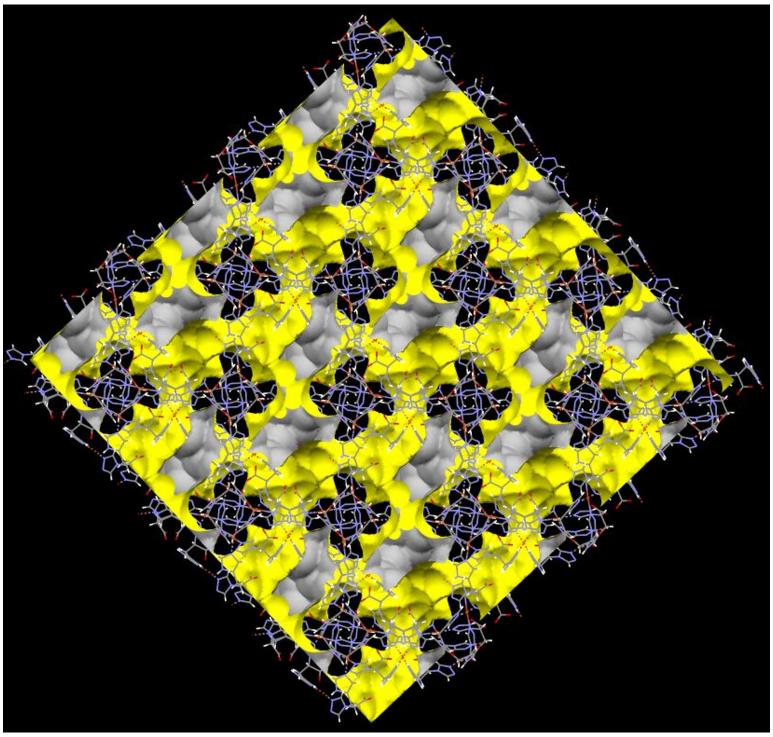

Figure S2. Solvent accessible surface of **TAMOF-1** calculated with a probe radius of 1.2 Å, showing the 3D intersected channels.

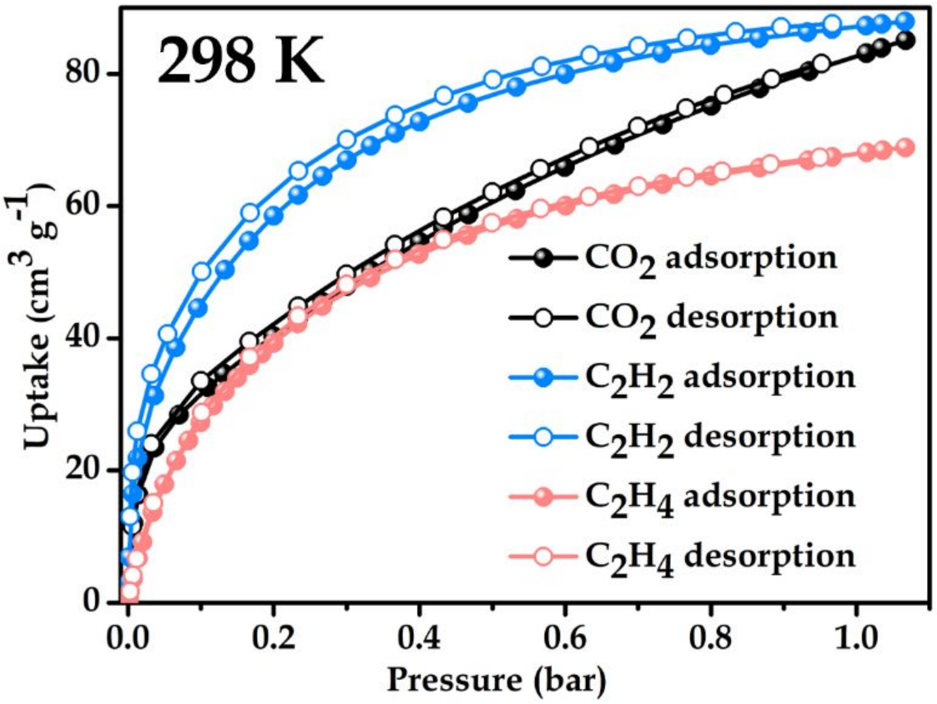

Figure S3. Single-component adsorption and desorption isotherms of C_2H_2, C_2H_4, and CO_2 at 298 K for **TAMOF-1**.

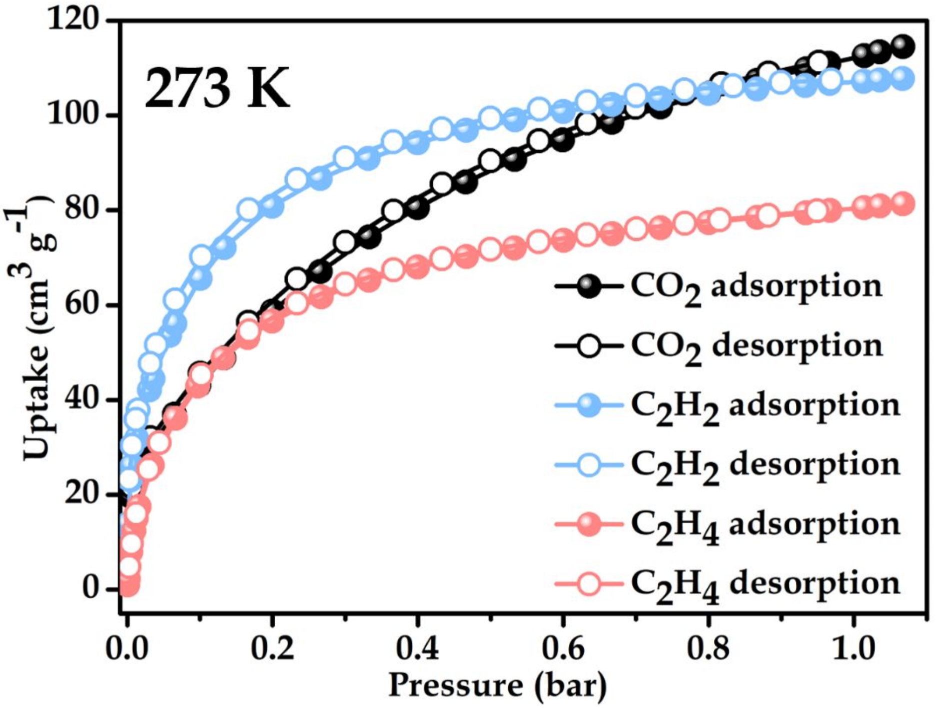

Figure S4. Single-component adsorption and desorption isotherms of C_2H_2, C_2H_4, and CO_2 at 273 K for **TAMOF-1**.

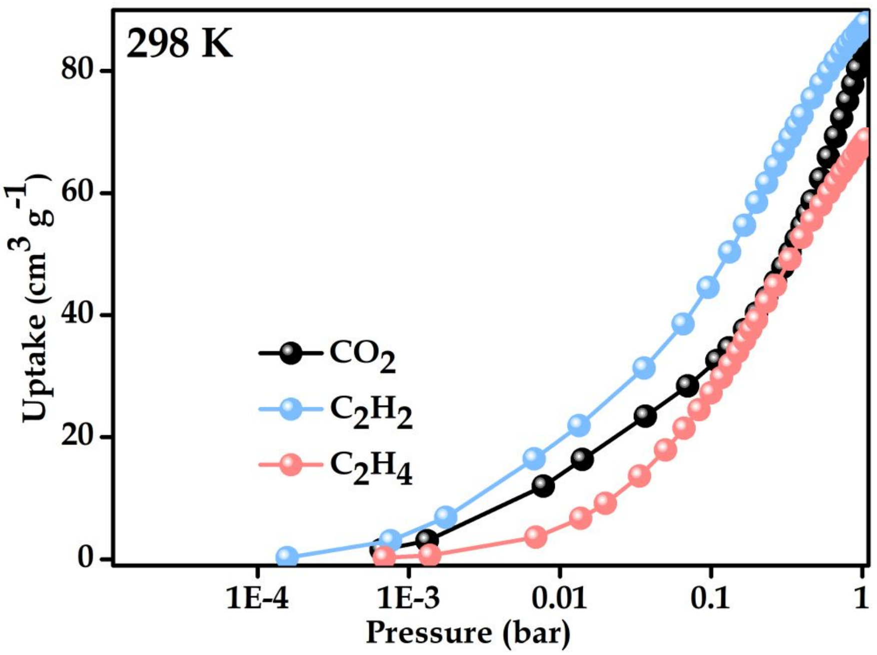

Figure S5. Adsorption isotherms of C_2H_2, C_2H_4, and CO_2 at 298 K for **TAMOF-1**.

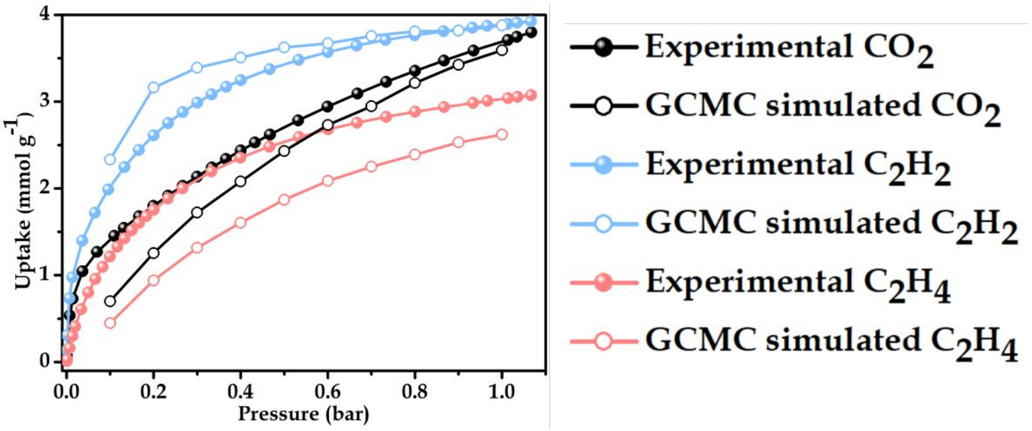

Figure S6. Comparison of GCMC-simulated and experimental isotherms of C_2H_2, C_2H_4, and CO_2 at 298 K.

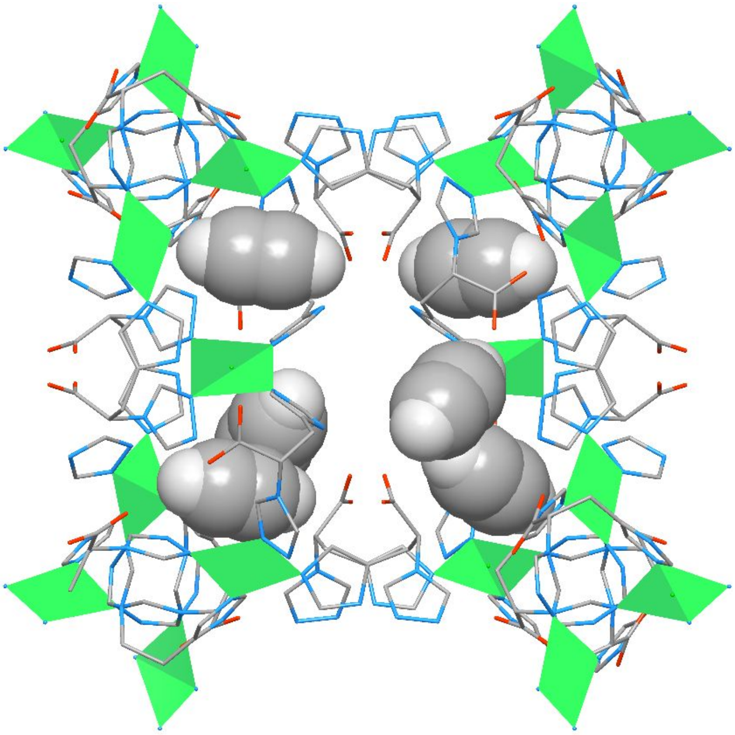

Figure S7. GCMC simulated adsorption of C_2H_2 in **TAMOF-1** at 0.1 bar.

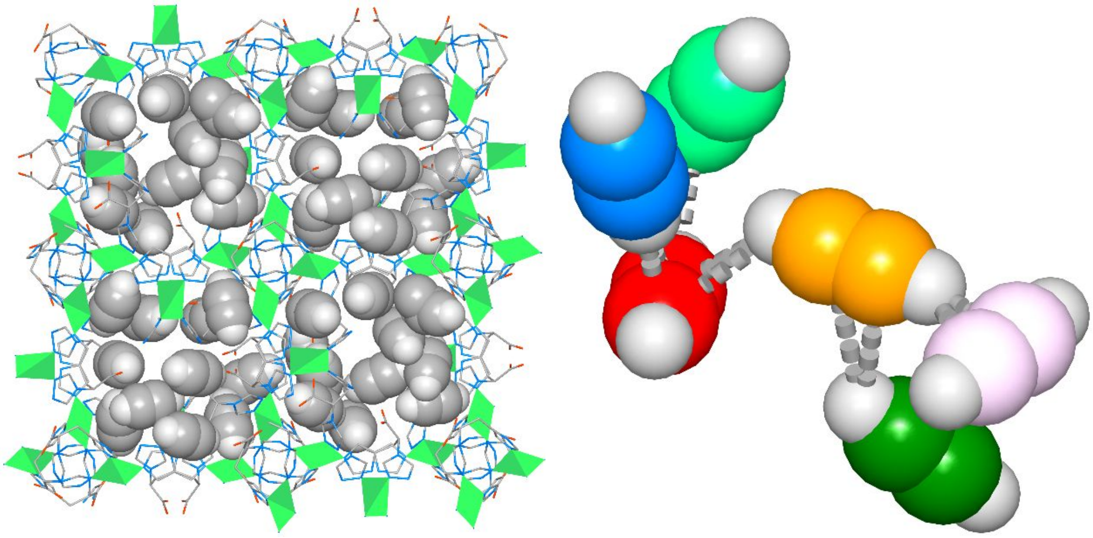

Figure S8. GCMC simulated adsorption of C_2H_2 in **TAMOF-1** at 1 bar (left) and the intermolecular interactions between C_2H_2 molecules (right).

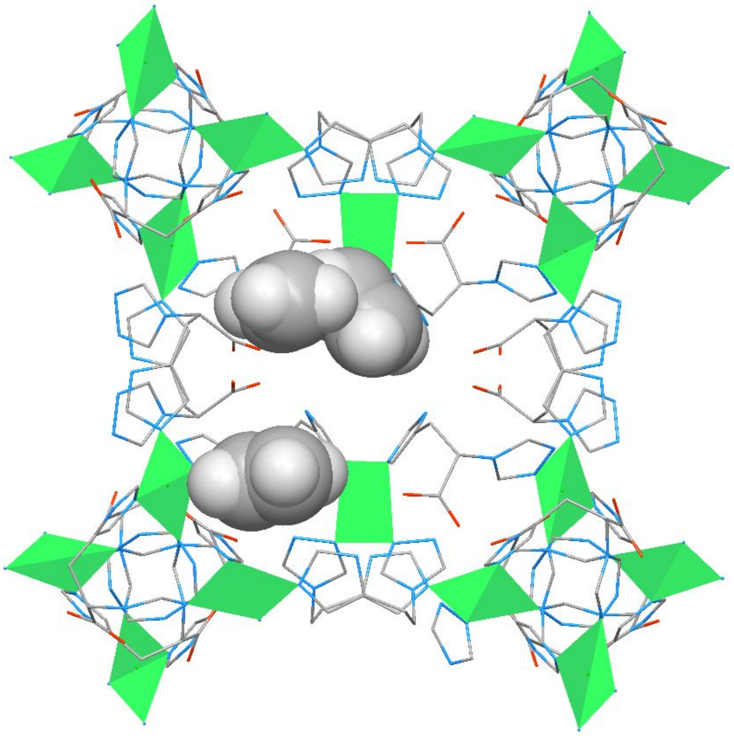

Figure S9. GCMC simulated adsorption of C_2H_4 in **TAMOF-1** at 0.1 bar.

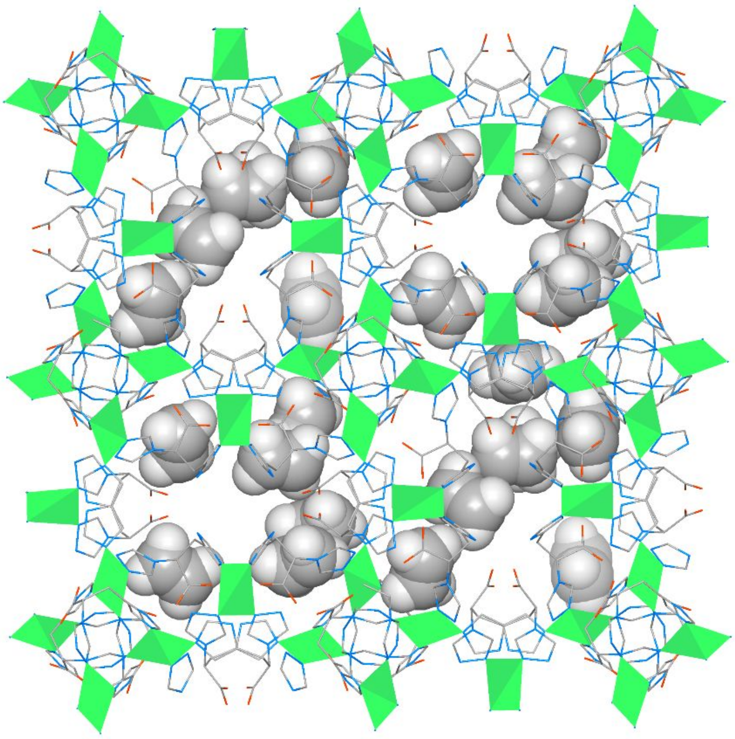

Figure S10. GCMC simulated adsorption of C_2H_4 in **TAMOF-1** at 1 bar.

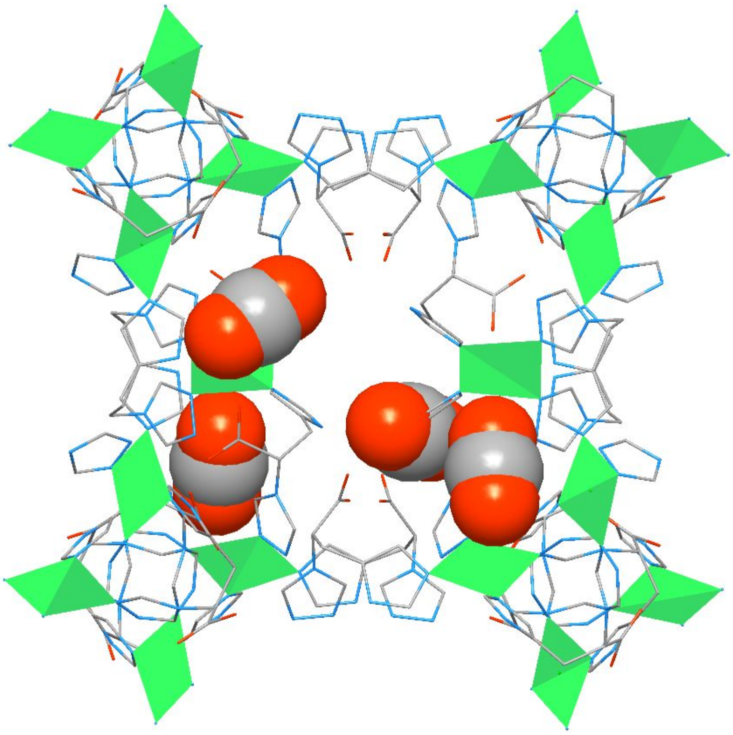

Figure S11. GCMC simulated adsorption of CO_2 in **TAMOF-1** at 0.1 bar.

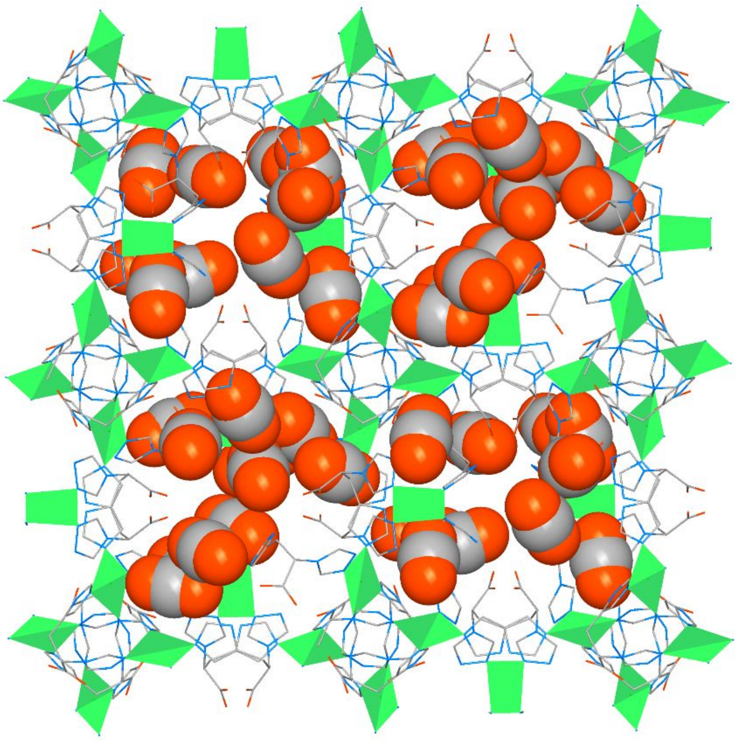

Figure S12. GCMC simulated adsorption of CO_2 in **TAMOF-1** at 1 bar.

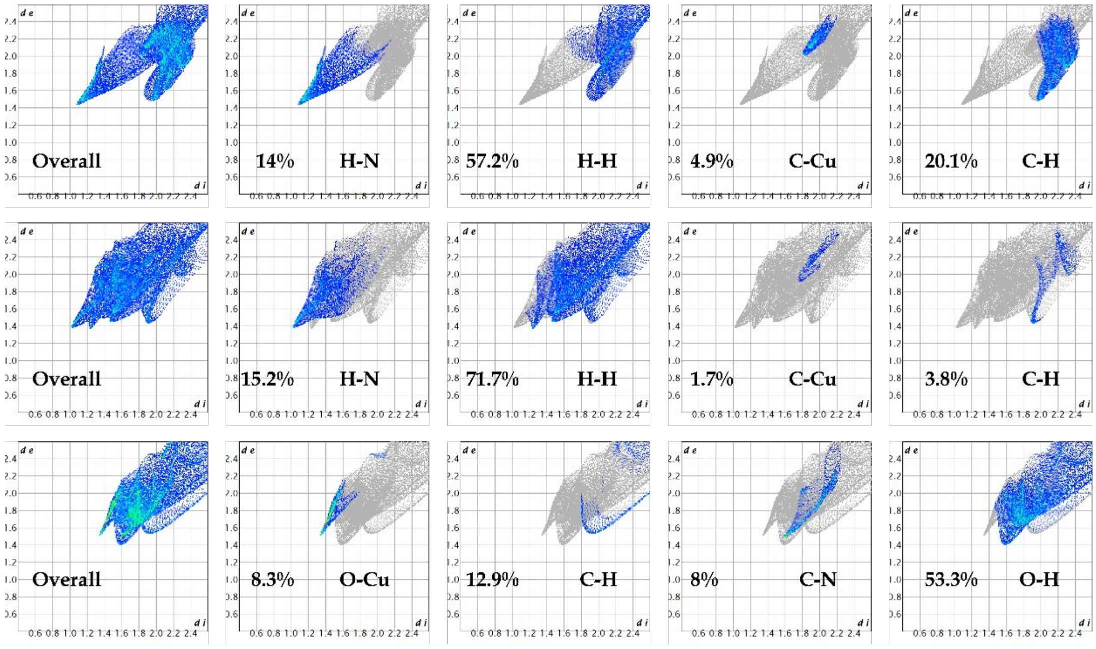

Figure S13. Derived 2D fingerprint plot of host-guest interactions in **TAMOF-1**. Top: C_2H_2; middle: C_2H_4; down: CO_2.

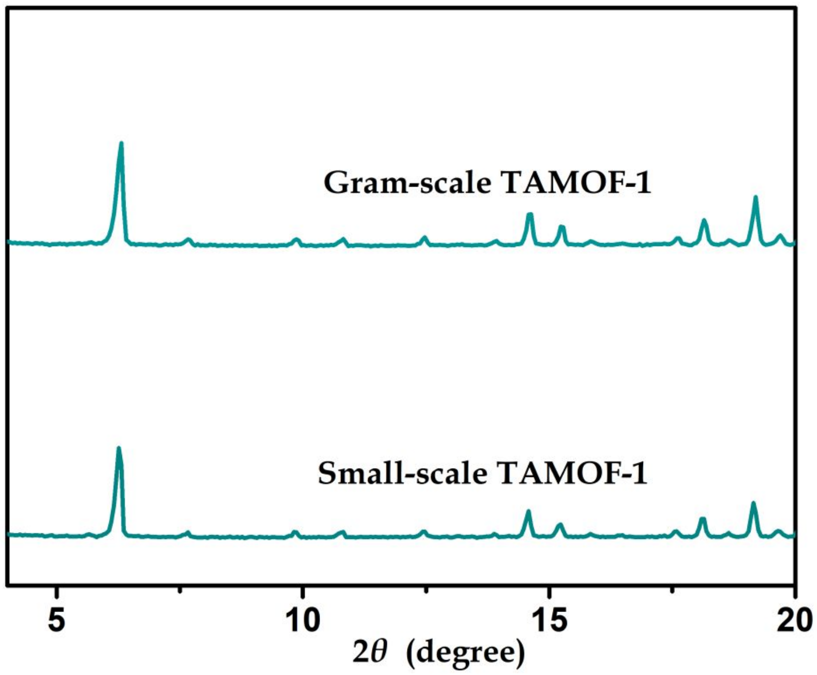

Figure S14. Comparison of PXRD patterns of **TAMOF-1** synthesized with different scale.

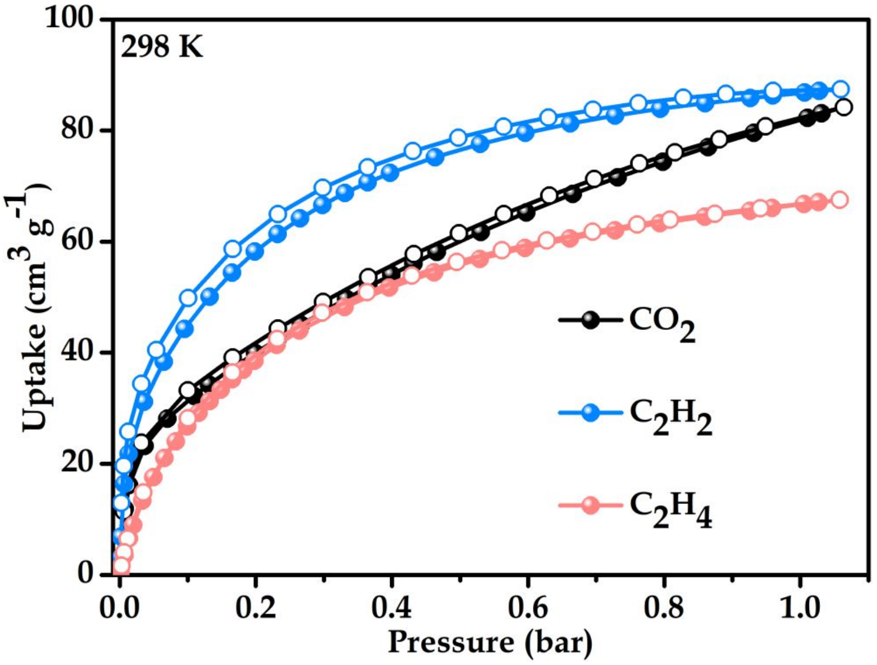

Figure S15. Second cycle single-component isotherms for **TAMOF-1.**

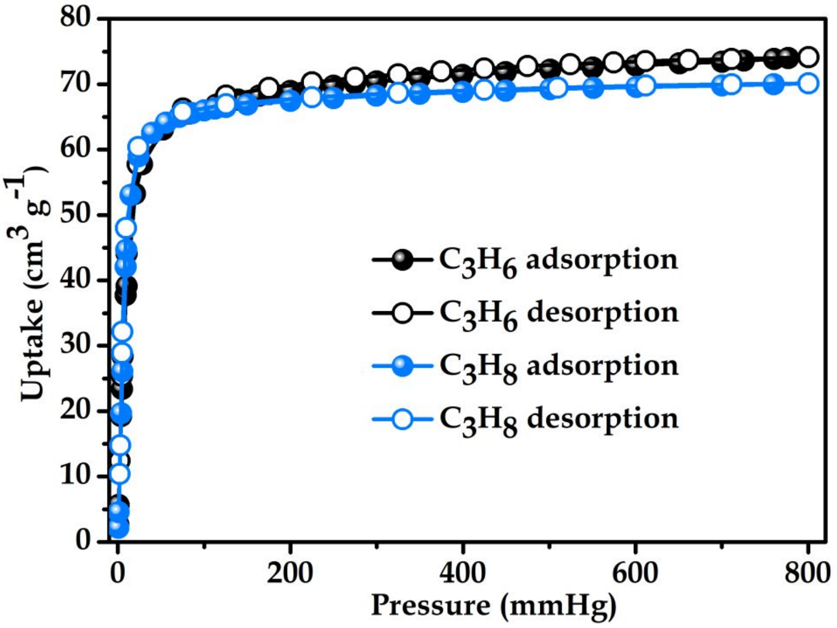

Figure S16. Single-component isotherms of C_3H_6 and C_3H_8 for **TAMOF-1** at 298 K.

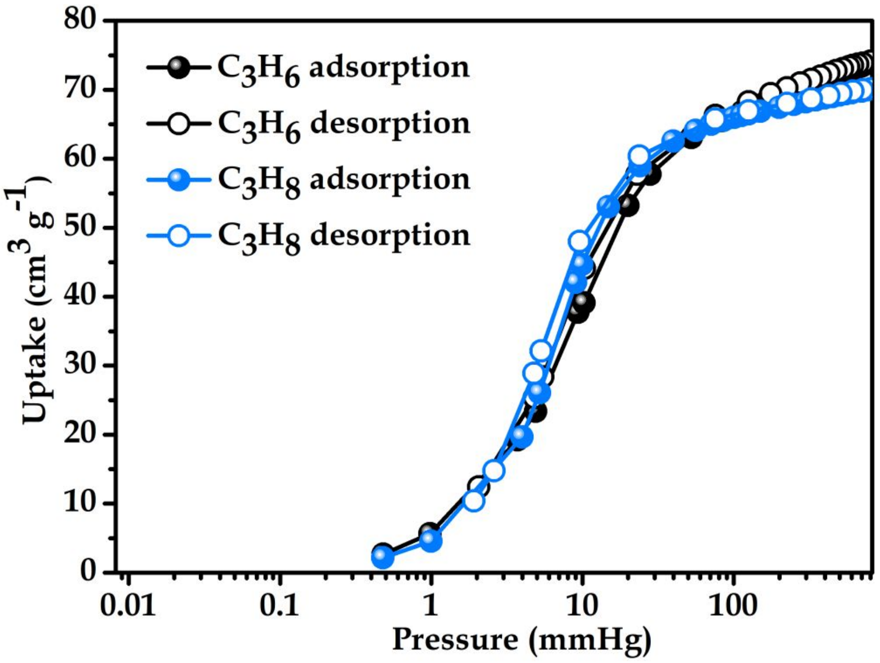

Figure S17. Single-component isotherms of C_3H_6 and C_3H_8 for **TAMOF-1** at 298 K.

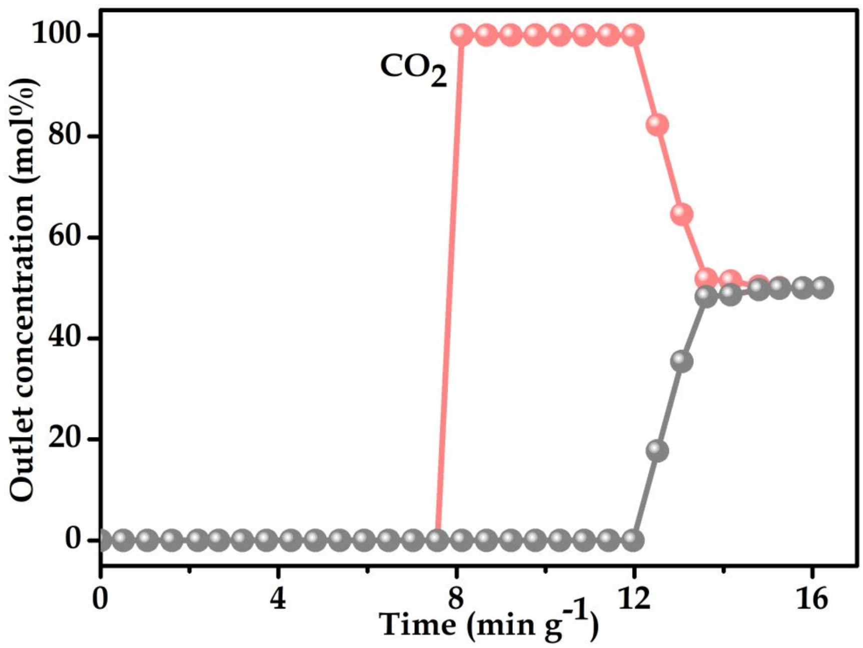

Figure S18. Second cycle breakthrough curves of **TAMOF-1** in binary C_2H_2/CO_2 mixture.

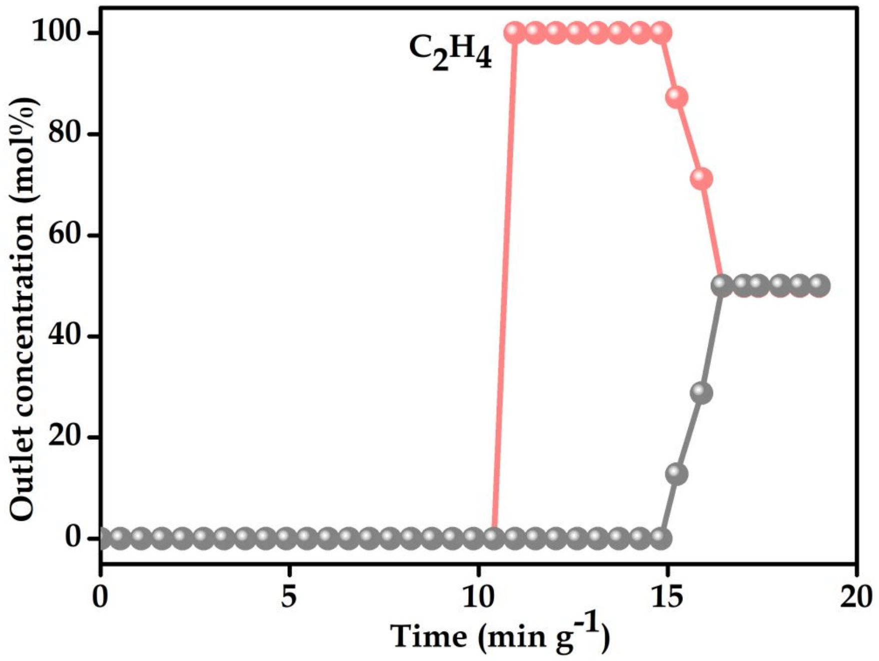

Figure S19. Second cycle breakthrough curves of **TAMOF-1** in binary C_2H_2/C_2H_4 mixture.

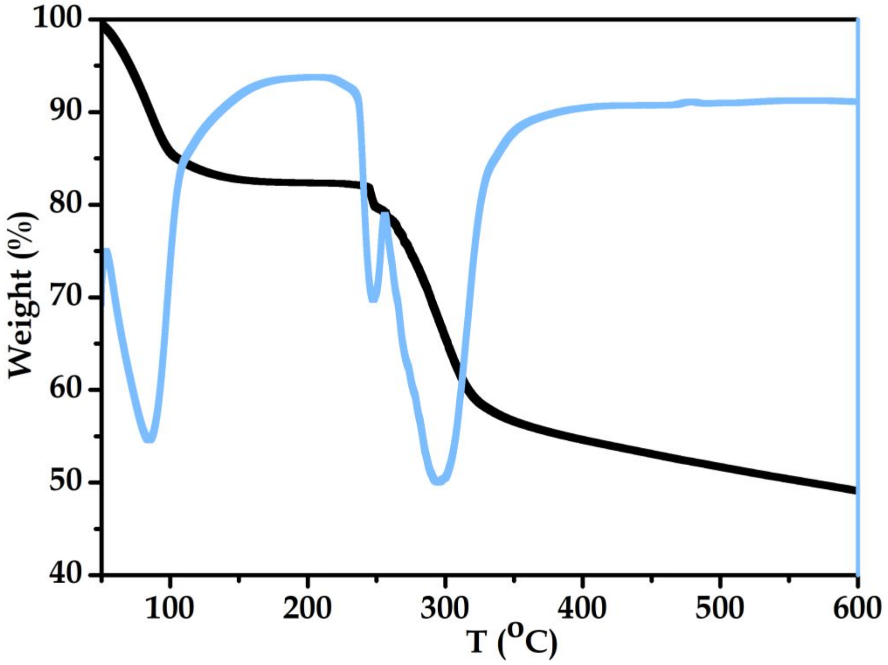

Figure S20. TGA curve of **TAMOF-1**

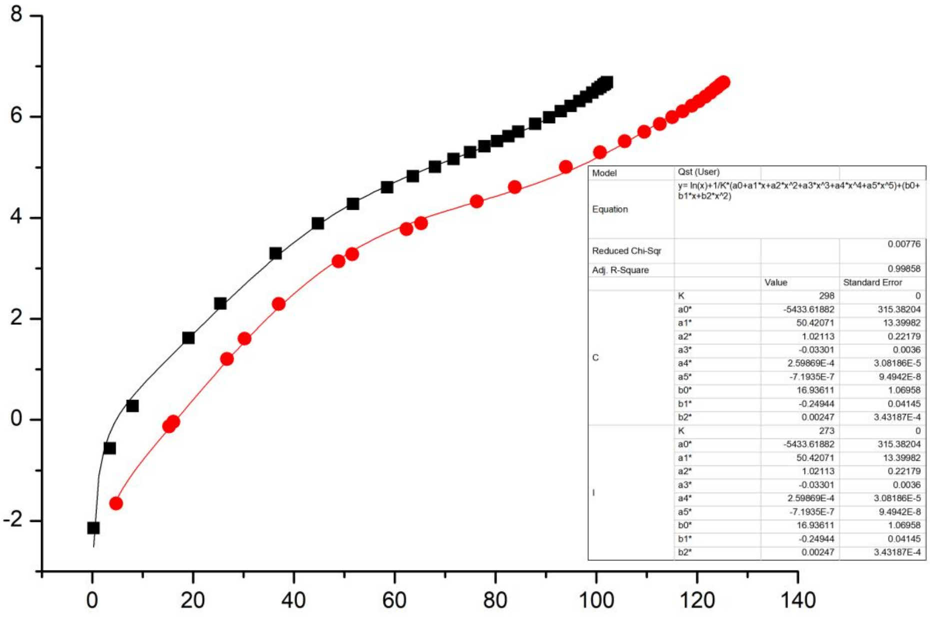

Figure S21. The fitting results of Q_{st} for C_2H_2 in **TAMOF-1** by using adsorption isotherms at 273 K and 298 K.

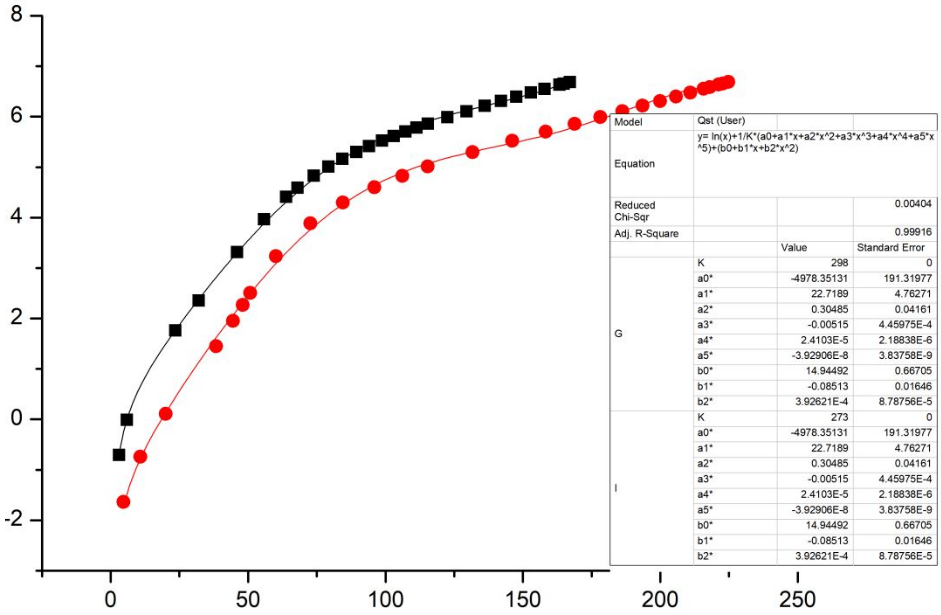

Figure S22. The fitting results of Q_{st} for CO_2 in **TAMOF-1** by using adsorption isotherms at 273 K and 298 K.

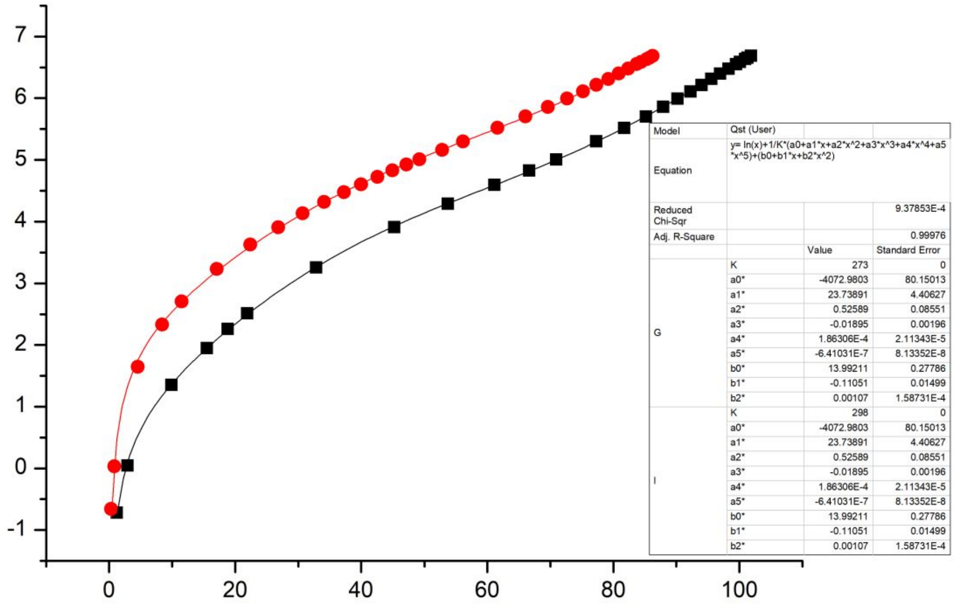

Figure S23. The fitting results of Q_{st} for C_2H_4 in **TAMOF-1** by using adsorption isotherms at 273 K and 298 K.

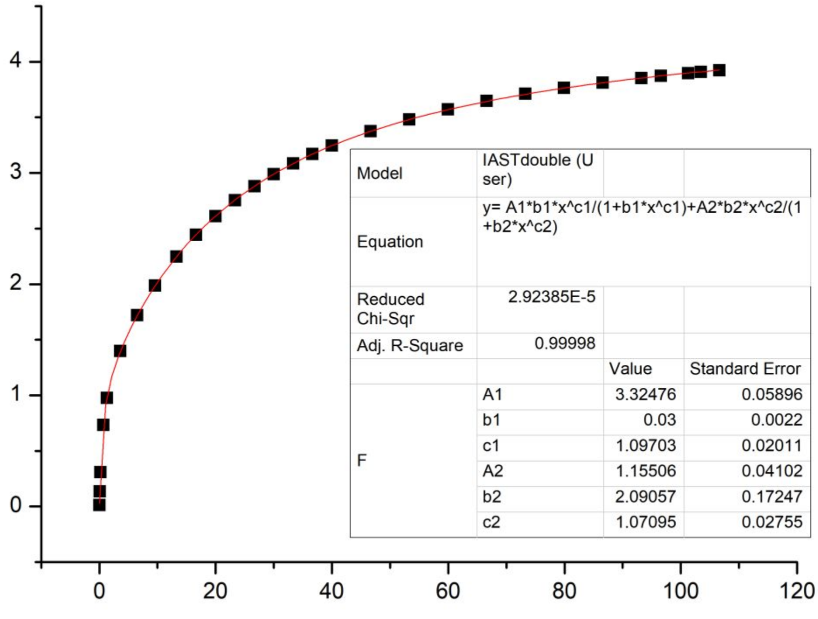

Figure S24. The fitting results of C_2H_2 isotherm at 298 K for TAMOF-1.

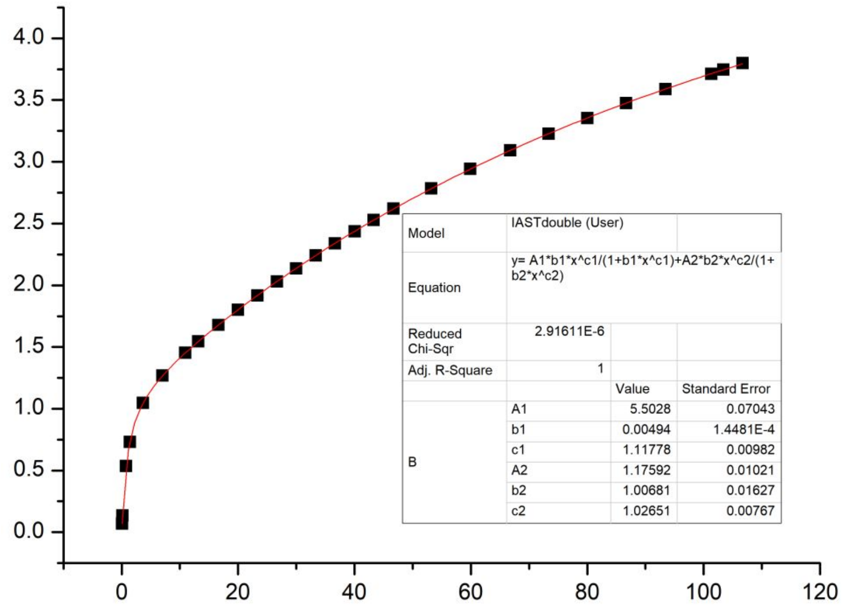

Figure S25. The fitting results of CO_2 isotherm at 298 K for TAMOF-1.

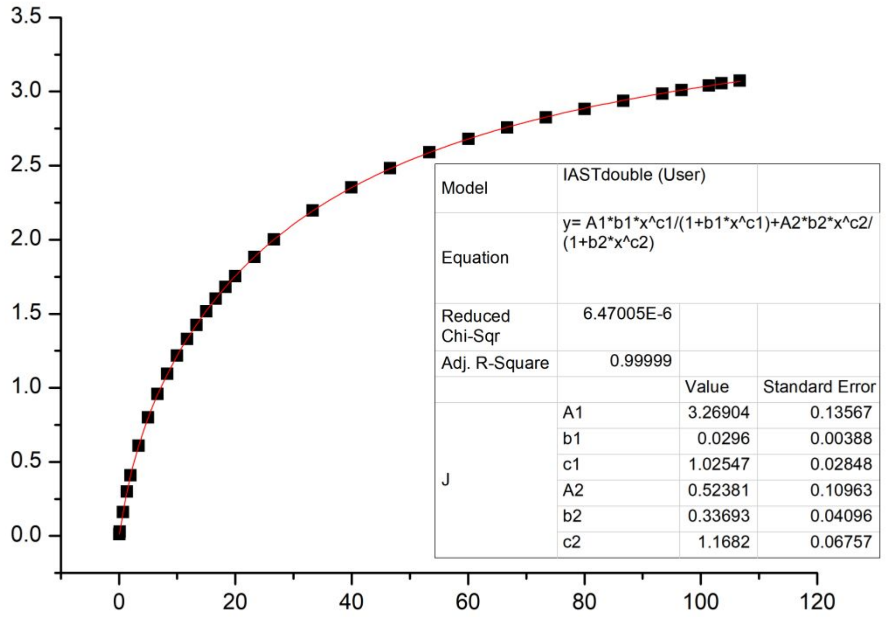

Figure S26. The fitting results of C_2H_4 isotherm at 298 K for TAMOF-1.

References

(1). S. J. Clark.; M. D. Segall.; C. J. Pickard.; P. J. Hasnip.; M. I. J. Probert.; K. Refson and M. C. Payne. *Z. Kristallogr.*, **2005**, *220*, 567.

(2). J. P. Perdew.; K. Burke.; M. Ernzerhof. *Phys. Rev. Lett*. **1996**, *77*, 3865.

(3). Vanderbilt, D. *Phys. Rev. B*. **1990**, *41*, 7892.

(4). Eguchi, R.; Uchida, S.; Mizuno, N., *Angew. Chem. Int. Ed.* **2012**, *51*, 1635.
